# Newly diagnosed diabetes based on an oral glucose tolerance test predicts cardiovascular outcomes in patients with coronary artery disease: An observational study

**DOI:** 10.1097/MD.0000000000029557

**Published:** 2022-07-15

**Authors:** Wei-Lin Chen, Wayne Huey-Herng Sheu, Yu-Hsuan Li, Jun-Sing Wang, Wen-Jane Lee, Kae-Woei Liang, Wen-Lieng Lee, I-Te Lee

**Affiliations:** a Division of Endocrinology and Metabolism, Department of Internal Medicine, Taichung Veterans General Hospital, Taichung, Taiwan; b Division of Endocrinology and Metabolism, Department of Internal Medicine, Taipei Veterans General Hospital, Taipei, Taiwan; c School of Medicine, National Yang Ming Chiao Tung University, Taipei, Taiwan; d Department of Computer Science & Information Engineering, National Taiwan University, Taipei, Taiwan; e Department of Medical Research, Taichung Veterans General Hospital, Taichung, Taiwan; f Cardiovascular Center, Taichung Veterans General Hospital, Taichung, Taiwan; g School of Medicine, Chung Shan Medical University, Taichung, Taiwan.

**Keywords:** angina, diabetes, oral glucose tolerance test, 2-hour postload glucose

## Abstract

Diabetes is prevalent in patients with coronary artery disease (CAD). Using the oral glucose tolerance test (OGTT), abnormal glucose regulation can be detected early in CAD patients without known diabetes. In the present study, we assessed the impact of abnormal glucose regulation on the long-term cardiovascular outcomes of patients with established CAD.

Patients hospitalized for a scheduled angiography due to angina were enrolled in Taichung Veterans General Hospital. Fasting plasma glucose (FPG) and 2-hour postload glucose (2hPG) were assessed using the OGTT. Hemoglobin A1c (HbA1c) and other biochemical analyses were assessed using fasting blood samples.

During a median follow-up period of 4.6 years, a composite of all-cause mortality, nonfatal myocardial infarction, and nonfatal stroke was recorded as the primary endpoint. In 682 enrolled patients who completed the follow-up, there were 16 myocardial infarction events, 12 stroke events, and 58 deaths as composite endpoints. According to FPG and 2hPG, patients with newly diagnosed diabetes had a 2-fold higher risk for the composite endpoint than those in the normal glucose group (hazard ratio [HR], 2.011; 95% confidence interval (CI), 1.101–3.673; *P* = .023); however, prediabetes was not significantly associated with the composite endpoint (HR, 1.452; 95% CI, 0.788–2.675; *P* = .232). On the other hand, patients with diabetes diagnosed by FPG and HbA1c did not have a significantly higher risk for the composite endpoint than those in the normal glucose group (HR, 1.321; 95% CI, 0.686–2.545; *P* = .405). A 2hPG ≥7.8 mmol/L was a significant predictor for the composite endpoint (odds ratio, 1.743; 95% CI, 1.060–2.863; *P* = .028) after adjusting for age, sex, and estimated glomerular filtration rate.

Diabetes, but not prediabetes, detected via OGTT is associated with a significantly increased risk for the composite endpoint in patients with established CAD. The 2hPG provided a greater predictive power for the composite endpoint than fasting glucose and HbA1c.

## 1. Introduction

Abnormal glucose regulation, including impaired fasting glucose, impaired glucose tolerance, and type 2 diabetes, is prevalent worldwide.^[1,2]^ In addition to newly diagnosed diabetes, impaired glucose tolerance has been found to be associated with an increased risk of all-cause mortality during 7 years of follow-up in European patients^[3]^ and approximately 5 years of follow-up in Asian patients.^[4]^ A high proportion of abnormal glucose regulation was particularly reported in patients with coronary artery disease (CAD), even if the patients had not previously been diagnosed with diabetes.^[5–7]^ Based on the European Society of Cardiology guidelines, developed in collaboration with the European Association for the Study of Diabetes, the oral glucose tolerance test (OGTT) is highly recommended for early diabetes detection in patients who have CAD.^[8]^

Although previously known diabetes predicted high mortality and recurrence of myocardial infarction (MI) in a previous Swedish study of 1062 patients, newly diagnosed abnormal glucose regulation, including prediabetes and diabetes, during acute coronary syndrome did not significantly predict all-cause mortality within 3 years of follow-up.^[9]^ In patients with acute MI enrolled in Poland, newly diagnosed diabetes identified by predischarge OGTT was significantly associated with all-cause but not major adverse cardiovascular events.^[10]^ Similarly, in the 1-year outcome report of the Euro Heart Survey,^[11]^ patients with newly diagnosed diabetes identified by OGTT were predicted to have a 2-fold and significantly higher mortality rate than those with normal glucose regulation. However, newly diagnosed diabetes only predicted a 30% increased risk of the composite endpoint of all-cause mortality, MI, and stroke, which was not significantly different from that associated with normal glucose regulation. The short-term impact of newly diagnosed diabetes by OGTT screen might be significant on total mortality but not revealed on cardiovascular events in Caucasian patients with CAD.^[11]^

It has been reported that Asians have higher glucose increments than Caucasians in response to the same food,^[12]^ and postprandial glucose provided a greater contribution to hyperglycemia in Asians than in Caucasians.^[13,14]^ We hypothesized that a high cardiovascular risk is significantly associated with newly diagnosed diabetes in patients who had undergone OGTT in a stable condition after an elective angiography for angina. We also examined the impact of prediabetes on the composite risk of all-cause mortality, nonfatal MI, and nonfatal stroke.

## 2. Materials and Methods

### 2.1. Patients and procedures

This prospective observational study was conducted in Taichung Veterans General Hospital between April 2009 and July 2017. The inclusion criteria were adult patients admitted for elective angiography due to angina and an established CAD meeting ≥ 1 of the following criteria: a history of MI, a history of coronary revascularization, or coronary lesion with lumen narrowing of ≥50% on angiography. The exclusion criteria were acute MI less than 4 weeks before hospitalization, known diabetes, fasting plasma glucose (FPG) ≥7.0 mmol/L (126 mg/dL) during hospitalization, acute or chronic infectious diseases, and severe systemic disease, such as end-stage renal disease, malignancies, autoimmune diseases, and psychiatric disorders. We also excluded patients who were pregnant. All candidates in a stable condition were enrolled during hospitalization and scheduled for an OGTT during a later outpatient visit. The study complied with the Declaration of Helsinki and was approved by the Institutional Review Board of Taichung Veterans General Hospital. Written consent was obtained from each patient before the study procedures began.

### 2.2. Procedure

Before discharge, patients were informed about the necessary preparation for the OGTT. After an overnight fasting period, blood samples were obtained before the OGTT and at 120 minutes during the OGTT at the outpatient visit. Fasting blood samples were collected and prepared to measure glucose levels, hemoglobin A1c (HbA1c), lipid profiles, and creatinine levels. In addition, blood samples were collected at 120 minutes for the 2-hour postload glucose (2hPG).

We followed up on patients by monitoring their electronic medical records in our hospital for the first episode of all-cause mortality, nonfatal MI, or nonfatal stroke after baseline assessment. Between January 2018 and March 2018, we arranged follow-ups by telephone for all the enrolled patients without an endpoint record before December 31, 2017. The first occurrence of all-cause mortality, nonfatal MI, or nonfatal stroke was recorded based on the statement of the patients themselves or their immediate family members.

### 2.3. Laboratory measurements

Plasma glucose levels were determined using the oxidase-peroxidase method (Wako Diagnostics, Tokyo, Japan). HbA1c was determined using cation-exchange high-performance liquid chromatography (NGSP certified; G8, TOSOH, Tokyo, Japan). Serum creatinine and lipid levels were determined using commercial kits (Beckman Coulter, Fullerton, USA). The estimated glomerular filtration rate (eGFR) was calculated according to the Modification of Diet in Renal Disease equation as follows: 186 × (serum creatinine [mg/dl])^−1.154^ × (age [year])^−0.203^ (×0.742, if female).^[15]^

Blood glucose regulation status was defined based on FPG and 2hPG levels. Normal glucose regulation was defined as FPG < 5.6 mmol/L (100 mg/dL) and 2hPG < 7.8 mmol/L (140 mg/dL). Newly diagnosed diabetes was defined as FPG ≥ 7.0 mmol/L or 2hPG ≥ 11.1 mmol/L (200 mg/dL). The prediabetes group was defined as patients with a glucose status between the normal glucose regulation and diabetes criteria.^[16]^

### 2.4. Statistical analysis

We present the mean ± standard deviation for continuous variables and numbers with percentages for categorical data. The clinical variables were tested for statistically significant differences using Student’s *t* tests for continuous variables between 2 groups, one-way analysis of variance tests for continuous variables among more than 2 groups, and χ^2^ tests for categorical variables. Triglycerides and eGFR were log-transformed in analyses due to skewed distributions. The first occurrence of all-cause mortality, nonfatal MI, or nonfatal stroke served as the primary composite endpoint. The univariate cumulative risk for the endpoint was assessed by Kaplan-Meier analysis and the overall significance was tested by the log-rank test. Multivariate Cox proportional hazards regression analyses were used to determine the primary endpoint according to glucose status. A 2-tailed *P* value of <.05 was considered statistically significant. Statistical analysis was performed using SPSS 22.0 (IBM, Armonk, NY).

## 3. Results

### 3.1. Baseline characteristics

**Figure 1.**
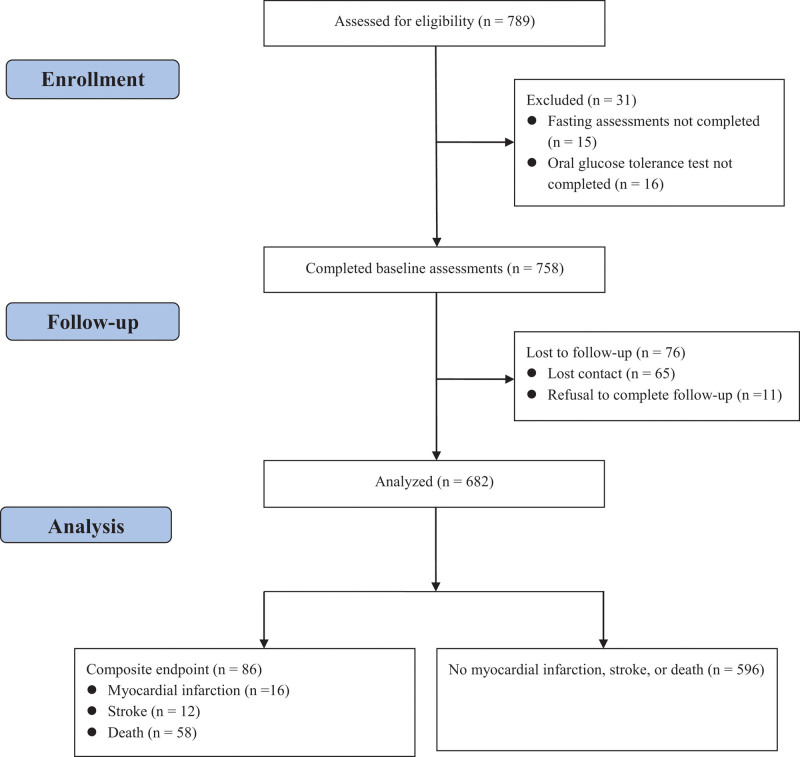
Flow diagram of the enrollment process for study subjects with coronary artery disease.

Among the 789 patients with established CAD, 758 patients completed the baseline assessments with the OGTT. There were 682 patients who completed the follow-up (Fig. 1). The baseline characteristics of the enrolled patients are shown in Table 1. Other than a higher serum high-density lipoprotein cholesterol (*P* = .008), lower triglycerides (*P* = .049), a lower proportion of hypertension (*P* = .003), and less use of antihypertensive drugs (*P* = .017) in the patients lost to follow-up, there were no significant differences at baseline between the patients lost to follow-up and those who completed the follow-up.

**Table 1:** The characteristics of the enrolled subjects (patients who completed the follow-up were categorized by FPG and 2hPG).

Patient characteristics	All enrolled patients (n = 758)			Patients who completed the follow-up (n = 682)			
	Lost to follow-up (n = 76)	Followed up (n = 682)	*P*	Normal glucose (n = 206)	Prediabetes (n = 292)	Diabetes (n = 184)	*P*
	Mean ± SD	Mean ± SD		Mean ± SD	Mean ± SD	Mean ± SD	
Age (yr)	64 ± 12	62 ± 12	.063	58 ± 12	63 ± 11*	64 ± 12*	<.001
Male, n (%)	67 (88.2)	621 (91.1)	.408	188 (91.3)	270 (92.5)	163 (88.6)	.350
Current smoking, n (%)	10 (13.2)	82 (12.0)	.774	29 (14.1)	28 (9.6)	25 (13.6)	.237
BMI (kg/m^2^)	25.4 ± 3.3	26.0 ± 3.5	.133	25.4 ± 3.5	26.0 ± 3.3	26.6 ± 3.6*	.005
Waist circumference (cm)	90.8 ± 8.1	91.1 ± 8.5	.728	89.3 ± 8.4	91.4 ± 8.1*	92.7 ± 8.8*	<.001
Systolic BP (mmHg)	128 ± 18	128 ± 18	.813	125 ± 17	128 ± 18	130 ± 18*	.005
Diastolic BP (mmHg)	74 ± 11	74 ± 10	.618	74 ± 11	75 ± 10	73 ± 10	.050
FPG (mmol/L)	5.4 ± 0.7	5.4 ± 0.9	.941	5.0 ± 0.3	5.3 ± 0.5*	6.1 ± 1.4^*,†^	<.001
2hPG (mmol/L)	8.7 ± 3.0	8.6 ± 3.1	.712	6.0 ± 1.2	8.2 ± 1.7*	12.0 ± 3.2^*,†^	<.001
HbA1c (%)	6.1 ± 0.6	6.0 ± 0.6	.212	5.6 ± 0.4	5.8 ± 0.4*	6.5 ± 0.8^*,†^	<.001
Triglyceride (mmol/L)‡	1.5 ± 1.0	1.7 ± 1.2	.049	1.6 ± 1.1	1.6 ± 1.2	1.9 ± 1.2†	.009
Total cholesterol (mmol/L)	4.5 ± 1.0	4.5 ± 1.1	.791	4.5 ± 1.2	4.4 ± 1.0	4.6 ± 1.0	.107
HDL cholesterol (mmol/L)	1.3 ± 0.4	1.1 ± 0.3	.008	1.2 ± 0.3	1.2 ± 0.3	1.1 ± 0.3	.553
LDL cholesterol (mmol/L)	2.8 ± 0.9	2.8 ± 0.9	.489	2.9 ± 1.0	2.7 ± 0.9*	2.7 ± 0.9	.035
eGFR (mL/min/1.73 m^2^)‡	72 ± 18	78 ± 23	.097	81 ± 22	79 ± 22	74 ± 23^*,†^	.005
Hypertension, n (%)	67 (88.2)	654 (95.9)	.003	193 (93.7)	281 (96.2)	180 (97.8)	.112
Antihypertensive agents, n (%)	63 (82.9)	623 (91.3)	.017	183 (88.8)	269 (92.1)	171 (92.9)	.293
ACE inhibitor or ARB, n (%)	51 (67.1)	447 (65.5)	.785	140 (68.0)	182 (62.3)	125 (67.9)	.311
α-blocker, n (%)	4 (5.3)	46 (6.7)	.622	11 (5.3)	20 (6.8)	15 (8.2)	.540
β-blocker, n (%)	15 (19.7)	154 (22.6)	.572	34 (16.5)	73 (25.0)*	47 (25.5)*	.044
Calcium channel blocker, n (%)	32 (42.1)	361 (52.9)	.073	95 (46.1)	164 (56.2)	102 (55.4)	.063
Diuretics, n (%)	9 (11.8)	98 (14.4)	.548	27 (13.1)	38 (13.0)	33 (17.9)	.272
Antiplatelet, n (%)	73 (96.1)	667 (97.8)	.342	200 (97.1)	286 (97.9)	181 (98.4)	.673
Statins, n (%)	50 (65.8)	513 (75.2)	.074	149 (72.3)	229 (78.4)	135 (73.4)	.238
Number of coronary arteries with stenosis, n (%)							
≤1	33 (43.4)	356 (52.2)	.343	110 (53.4)	152 (52.1)	94 (51.1)	.237
2	27 (35.5)	209 (30.6)		68 (33.0)	89 (30.5)	52 (28.3)	
3	16 (21.1)	117 (17.2)		28 (13.6)	51 (17.5)	38 (20.7)	
Composite endpoint§		86 (12.6)		16 (7.8)	31 (10.6)	39 (21.2)	<.001

ACE = angiotensin-converting enzyme, ARB = angiotensin II receptor blocker, BMI = body mass index, BP = blood pressure, eGFR = estimated glomerular filtration rate, FPG = fasting plasma glucose, HbA1c = hemoglobin A1c, HDL = high-density lipoprotein, LDL = low-density lipoprotein, 2hPG = 2-hour postload glucose.

* Indicates a significant difference from the normal glucose tolerance group.

† Indicates a significant difference from the prediabetes group.

‡ Triglycerides and eGFR are log-transformed in the analyses due to their skewed distributions.

§ The composite endpoint includes all-cause mortality, nonfatal myocardial infarction, and nonfatal stroke.

According to the baseline FPG and 2hPG, patients were divided into the following 3 groups: 206 (30.2%) patients were in the normal glucose group, 292 (42.8%) in the prediabetes group, and 184 (27.0%) in the diabetes group. The patients in the normal glucose group were younger than those in the prediabetes and diabetes groups. The patients in the normal glucose group had a lower body mass index than those in the diabetes group. The patients in the normal glucose group had a lower waist circumference than those in the prediabetes and diabetes groups. The patients in the normal glucose group had lower systolic blood pressure (BP) than those in the diabetes group. Glucose and HbA1c levels showed a significantly increasing trend from the normal glucose group to the diabetes group. The patients in the normal glucose group had a higher low-density lipoprotein (LDL) cholesterol level than those in the prediabetes group. The patients in the diabetes group had higher LDL cholesterol levels than those in the normal glucose and prediabetes groups. The patients in the normal glucose group had a lower proportion of using beta-blockers than those in the prediabetes and diabetes groups (Table 1).

### 3.2. Follow-up in patients grouped by FPG and 2hPG

**Figure 2.**
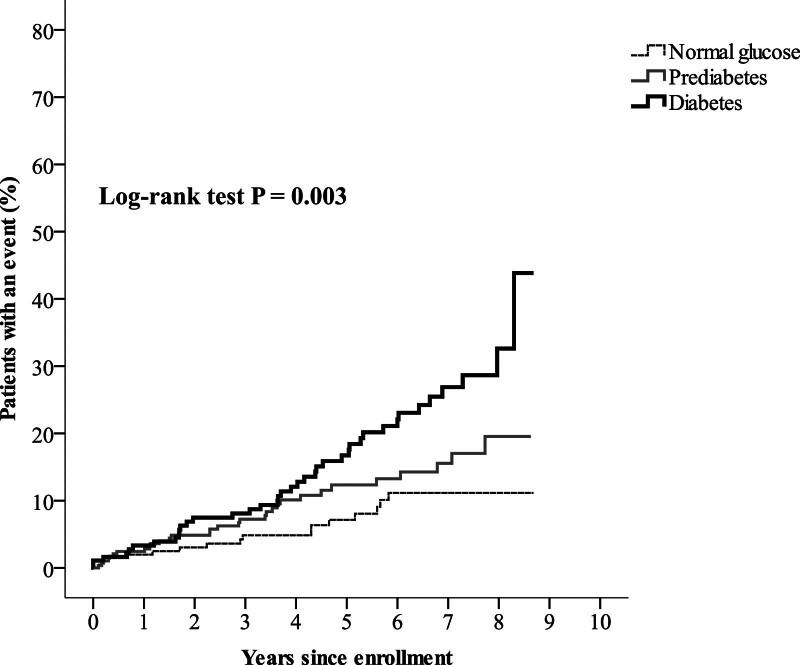
Kaplan-Meier curves showing the composite endpoint of all-cause mortality, nonfatal myocardial infarction (MI), and nonfatal stroke among the 3 different glucose regulation groups of patients with coronary artery disease.

During the median follow-up period of 4.6 years (interquartile range between 2.6 and 6.9 years), there were 16 MI events, 12 stroke events, and 58 deaths that occurred as the primary endpoint. The incidence of the composite endpoint was 2.75% among all follow-up patients, including 1.59% in the normal glucose group, 2.58% in the prediabetes group, and 4.22% in the diabetes group. As shown in Figure 2, the composite endpoint rate was significantly different among the 3 groups based on Kaplan-Meier analysis (log-rank test *P* = .003). To identify the risk for the composite endpoint among these 3 groups, multivariate Cox regression analysis was conducted (Table 2). The patients in the diabetes group showed a 2-fold higher risk for the composite endpoint than those in the normal glucose group (hazard ratio [HR], 2.011; 95% confidence interval (CI), 1.101–3.673; *P* = .023) after adjusting for sex, age, body mass index, systolic BP, eGFR, LDL cholesterol, triglycerides, and current use of beta-blockers.

**Table 2:** Effects of the associated risk factors on the composite endpoint of all-cause mortality, nonfatal myocardial infarction, and nonfatal stroke.

Variables	Patient number	Crude		Model 1		Model 2		Model 3	
		HR (95% CI)	*P*	HR (95% CI)	*P*	HR (95% CI)	*P*	HR (95% CI)	*P*
According to FPG and 2hPG									
Normal glucose	206	1.000		1.000		1.000		1.000	
Prediabetes	292	1.641 (0.897–3.001)	.108	1.414 (0.770–2.597)	.264	1.363 (0.740–2.511)	.320	1.452 (0.788–2.675)	.232
Diabetes	184	2.626 (1.467–4.699)	.001	2.154 (1.196–3.880)	.011	1.947 (1.067–3.553)	.030	2.011 (1.101–3.673)	.023
According to FPG and HbA1c									
Normal glucose	175	1.000		1.000		1.000		1.000	
Prediabetes	403	0.902 (0.508–1.602)	.726	0.885 (0.499–1.571)	.677	0.859 (0.482–1.532)	.607	0.833 (0.465–1.493)	.540
Diabetes	104	1.811 (0.963–3.406)	.065	1.609 (0.855–3.029)	.140	1.394 (0.729–2.667)	.316	1.321 (0.686–2.545)	.405
According to FPG, 2hPG and HbA1c									
Normal glucose	109	1.000		1.000		1.000		1.000	
Prediabetes	389	0.998 (0.465–2.140)	.995	0.927 (0.432–1.990)	.846	0.893 (0.414–1.930)	.774	0.894 (0.413–1.934)	.775
Diabetes	184	1.947 (0.907–4.180)	.087	1.627 (0.755–3.507)	.214	1.449 (0.660–3.178)	.355	1.445 (0.656–3.182)	.360

Model 1: adjustment for sex and age. Model 2: adjustment for sex, age, body mass index, and systolic blood pressure. Model 3: adjustment for sex, age, body mass index, systolic blood pressure, estimated glomerular filtration rate, low-density lipoprotein, triglycerides, and current use of beta-blockers.

CI = confidence interval, FPG = fasting plasma glucose, HbA1c = hemoglobin A1c, HR = hazard ratio, 2hPG = 2-hour postload glucose.

### 3.3. The effects of using HbA1c on endpoint prediction

To evaluate the performance of HbA1c on endpoint prediction, an HbA1c of < 5.7% belonged to normal glucose regulation, an HbA1c value between 5.7% and 6.4% belonged to prediabetes, and an HbA1c of ≥ 6.5% belonged to diabetes.^[16]^ There were 175 patients with normal glucose regulation defined by FPG < 5.6 mmol/L and HbA1c < 5.7%; 104 patients with diabetes defined by FPG ≥ 7.8 mmol/L or/and HbA1c ≥ 6.5%; and 403 patients with prediabetes. Neither diabetes nor prediabetes based on FPG and HbA1c was significantly associated with the occurrence of the endpoint (HR, 1.321; 95% CI, 0.686–2.545; and HR, 0.833; 95% CI, 0.465–1.493, respectively). Even if patients were grouped using HbA1c, FPG, and 2hPG, neither diabetes nor prediabetes was significantly associated with the occurrence of the endpoint (HR, 1.445; 95% CI, 0.656–3.182; and HR, 0.894; 95% CI, 0.413–1.934, respectively, Table 2).

**Figure 3.**
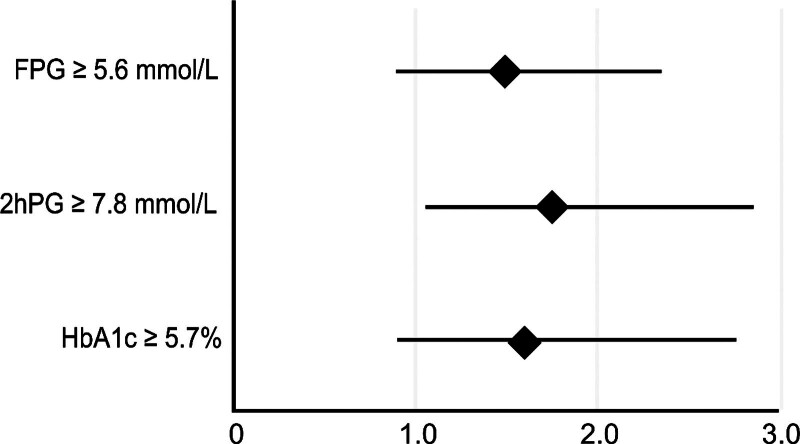
Odds ratio with a 95% confidence interval of the ability of the fasting plasma glucose (FPG), 2-hour postload glucose (2hPG), and hemoglobin A1c (HbA1c) levels to predict the composite endpoint in 682 patients with coronary artery disease but without a history of diabetes before the oral glucose tolerance test (after adjusting for age, sex, and estimated glomerular filtration rate).

An additional logistic regression analysis was conducted to assess the contribution of FPG, 2hPG, and HbA1c levels to the composite endpoint. A 2hPG ≥ 7.8 mmol/L (140 mg/dL) was shown to be a significant predictor for the composite endpoint (odds ratio [OR], 1.743; 95% CI, 1.060–2.863; *P* = .028) and had a stronger contribution than an FPG ≥ 5.6 mmol/L (OR, 1.457; 95% CI, 0.900–2.358; *P* = 0.126) and an HbA1c ≥ 5.7% (OR, 1.588; 95% CI, 0.903–2.791; *P* = .108) after adjusting for age, sex, and eGFR (Fig. 3).

## 4. Discussion

The main finding in the present study is that newly diagnosed diabetes based on OGTT predicted a significantly higher risk for the composite endpoint of all-cause mortality, nonfatal MI, and nonfatal stroke than normal glucose regulation in patients with stable CAD during a median follow-up of 4.8 years. In the Danish study of patients after acute MI with a follow-up of 9.8 years (median 74 months), newly diagnosed diabetes based on OGTT increased 56% risk of all-cause mortality.^[17]^ In the Swedish study of patients after acute MI with a follow-up of mean of 4.8 years, OGTT-diagnosed diabetes increased 9% risk of cardiovascular events after adjusting for age and sex.^[18]^ In both of the above studies, however, there was no significant difference in the long-term prognosis between OGTT-diagnosed diabetes and normal glucose regulation.^[17,18]^ Our study provides evidence that OGTT screening for newly diagnosed diabetes can predict long-term cardiovascular risk in Asian patients with CAD.

Prediabetes is associated with a state of chronic inflammation, oxidative stress, endothelial dysfunction, and hypercoagulation.^[19–21]^ Furthermore, prediabetes has been reported to be predictive of cardiovascular disease in a large-scale meta-analysis of the general population.^[22]^ A 60% higher risk of unrecognized MI was observed in patients with prediabetes than in patients with normal glucose regulation in a multiethnic population without previous cardiovascular disease.^[23]^ In the present study, however, prediabetes diagnosed based on OGTT showed a 45% increased risk for the composite endpoint, which was not significantly different from that associated with normal glucose regulation in patients with established CAD. In line with our results, several previous studies showed no significant difference in cardiovascular risk between patients with prediabetes and those with normal glucose regulation.^[9,11,17]^ In the recently reported Innovation to Reduce Cardiovascular Complications of Diabetes at the Intersection (ARTEMIS) study, prediabetes was associated with a lower risk of a major adverse cardiac event than known diabetes, and this was not significantly different from the risk in normal glucose regulation in the patients with established CAD.^[24]^ Based on our results, however, Asians with prediabetes seem to have a higher risk than Caucasians with prediabetes, which was reported to have an HR of 1.02 for the composite endpoint in the Euro Heart Survey^[11]^ and HR of 1.07 for all-cause mortality in the Danish study.^[17]^

Postprandial hyperglycemia is closely associated with cardiovascular risk, and transient high glucose levels might increase CAD risk more than chronic hyperglycemia.^[25]^ Based on a study using a hyperglycemic clamp, the levels of inflammatory cytokines increased after hyperglycemic pulses, which might result from an oxidative reaction.^[26,27]^ Ceriello et al^[28]^ also reported that the increased oxidative stress induced by hyperglycemic pulses was significantly associated with endothelial dysfunction. Several studies have reported that a high 2hPG is a strong predictor of cardiovascular risk in CAD patients without previously known diabetes.^[29–31]^ In the EUROpean Action on Secondary and Primary Prevention through Intervention to Reduce Events IV survey reported by Shahim et al,^[32]^ a 2hPG ≥ 7.8 mmol/L was associated with a 38% higher composite risk for cardiovascular mortality, nonfatal MI, stroke, or hospitalization for heart failure than a 2hPG < 7.8 mmol/L in CAD patients without a history of diabetes before the OGTT after a median follow-up of 2 years. In an Asian study reported by Kuramitsu et al,^[31]^ a 2hPG ≥ 7.8 mmol/L was associated with a 59% higher composite risk of cardiovascular mortality, nonfatal MI, stroke, or revascularization than normal glucose regulation in CAD patients without a history of diabetes before the OGTT after a median follow-up of 4.3 years. In our present study, a 2hPG ≥ 7.8 mmol/L also showed a 74% higher composite risk of cardiovascular mortality, nonfatal MI, and nonfatal stroke than normal glucose regulation in CAD patients without a history of diabetes before the OGTT. Therefore, a 2hPG ≥ 7.8 mmol/L is a stronger predictor for cardiovascular disease than either an FPG ≥ 6.1 mmol/L in the EUROpean Action on Secondary and Primary Prevention through Intervention to Reduce Events IV survey^[32]^ or an FPG ≥ 5.6 mmol/L in the present study.

In the present study, the rate of newly diagnosed diabetes based on the OGTT was 27%, which is similar to the rate of 26.4% in the China Heart Survey^[6]^ but higher than that (14%) in the Euro Heart Survey.^[5]^ Since there is a high proportion of diabetes diagnosed by OGTT and a high cardiovascular risk in Asian patients with established CAD and newly diagnosed diabetes, OGTT screening can be helpful for physicians to predict clinical prognoses and to set treatment goals.

The OGTT is important for the diagnosis of diabetes in patients with CAD because a large proportion of these patients have postprandial hyperglycemia.^[8]^ However, it is challenging to use the OGTT to diagnose diabetes. It has been suggested to use both the FPG and HbA1c levels to screen for diabetes;^[33]^ however, a previous study conducted in north Norway reported low sensitivity in the diagnosis of diabetes when using an HbA1c of ≥6.5%.^[34]^ In patients with CAD, in particular, diabetes might be underdiagnosed more often with an HbA1c assessment than with the OGTT.^[35,36]^ Karayiannides et al.^[18]^ reported that HbA1c predicted cardiovascular events in patients after acute MI, despite HbA1c having a lower propensity to diagnose diabetes than the OGTT. Moreover, among patients without known diabetes who experienced acute MI, the long-term mortality risk of patients with an HbA1c of ≥6.5% was similar to that of patients with an HbA1c of <6.5% and OGTT-diagnosed diabetes.^[17]^ Therefore, using the OGTT is still advantageous in predicting mortality risk for patients with CAD, even if their HbA1c is <6.5%. Notably, it has been reported that 2hPG, rather than HbA1c, is associated with the severity of CAD.^[36]^ The strength of the present study is that Asians who may have postprandial hyperglycemia were enrolled.

There are some limitations in the present study. First, we did not control the treatment for CAD during the follow-up period in this observational study. Second, we did not assess the 1-hour postload glucose (1hPG) in the present study. The 1hPG might be a better predictor for all-cause and cardiovascular mortality than the 2hPG.^[37]^ It has been reported that 1hPG reflects physiological changes in diabetes and is suitable for the prediction and diagnosis of diabetes.^[38,39]^ Therefore, both 1hPG and 2hPG are important in predicting long-term cardiovascular disease.^[40]^ Third, 2hPG was based on OGTT, and our results cannot, therefore, be applied to postprandial glucose. A postmeal hyperglycemic episode after breakfast was reported not to be predictive of cardiovascular events in patients with established CAD.^[41]^ Fourth, we did not assess changes in glucose during the follow-up. It has been reported that inhospital glucose variability after coronary intervention and outpatient glucose variability are associated with cardiovascular events and mortality.^[42,43]^ Finally, we did not know whether intensive glucose control would improve the composite endpoint in this observational study. Further studies evaluating the effects of early intensive treatment for patients with newly diagnosed diabetes and CAD are warranted.

In conclusion, diabetes can be detected early via OGTT screening in patients with established CAD. Newly diagnosed diabetes, but not prediabetes, is associated with a significantly higher risk for the composite endpoint, including all-cause mortality, nonfatal MI, and nonfatal stroke than normal glucose regulation in patients with CAD. The 2hPG provided a greater predictive power for the composite endpoint than the FPG in these CAD patients without a known history of diabetes.

## Acknowledgments

We thank the Biostatistics Task Force of Taichung Veterans General Hospital, Taichung, Taiwan, for statistical analysis.

## Author contributions

Conceptualization: Wei-Lin Chen, Wayne Huey-Herng Sheu, Yu-Hsuan Li, Jun-Sing Wang, Wen-Jane Lee, Kae-Woei Liang, Wen-Lieng Lee, I-Te Lee.

Data curation: Wei-Lin Chen, Yu-Hsuan Li, Jun-Sing Wang, Wen-Jane Lee, Kae-Woei Liang, I-Te Lee.

Formal analysis: I-Te Lee.

Funding acquisition: Wayne Huey-Herng Sheu, I-Te Lee.

Investigation: Wei-Lin Chen, Wayne Huey-Herng Sheu, Yu-Hsuan Li, Jun-Sing Wang, Wen-Jane Lee, Kae-Woei Liang, Wen-Lieng Lee, I-Te Lee.

Methodology: Wei-Lin Chen, Wayne Huey-Herng Sheu, Yu-Hsuan Li, Jun-Sing Wang, Wen-Jane Lee, Kae-Woei Liang, Wen-Lieng Lee, I-Te Lee.

Project administration: Wayne Huey-Herng Sheu, I-Te Lee.

Resources: Wayne Huey-Herng Sheu, I-Te Lee.

Software: Wen-Jane Lee.

Supervision: Wayne Huey-Herng Sheu, Wen-Lieng Lee, I-Te Lee.

Validation: I-Te Lee.

Visualization: Wei-Lin Chen, I-Te Lee.

Writing – original draft: Wei-Lin Chen.

Writing – review and editing: I-Te Lee.
